# An empirical comparison of deep learning explainability approaches for EEG using simulated ground truth

**DOI:** 10.1038/s41598-023-43871-8

**Published:** 2023-10-18

**Authors:** Akshay Sujatha Ravindran, Jose Contreras-Vidal

**Affiliations:** 1 Noninvasive Brain-Machine Interface System Laboratory, Department of Electrical and Computer Engineering, University of Houston, Houston, 77204 USA; 2 IUCRC BRAIN, University of Houston, Houston, 77204 USA; 3 Alto Neuroscience, Los Altos, CA 94022 USA

**Keywords:** Electroencephalography - EEG, Neural decoding, Machine learning

## Abstract

Recent advancements in machine learning and deep learning (DL) based neural decoders have significantly improved decoding capabilities using scalp electroencephalography (EEG). However, the interpretability of DL models remains an under-explored area. In this study, we compared multiple model explanation methods to identify the most suitable method for EEG and understand when some of these approaches might fail. A simulation framework was developed to evaluate the robustness and sensitivity of twelve back-propagation-based visualization methods by comparing to ground truth features. Multiple methods tested here showed reliability issues after randomizing either model weights or labels: e.g., the saliency approach, which is the most used visualization technique in EEG, was not class or model-specific. We found that DeepLift was consistently accurate as well as robust to detect the three key attributes tested here (temporal, spatial, and spectral precision). Overall, this study provides a review of model explanation methods for DL-based neural decoders and recommendations to understand when some of these methods fail and what they can capture in EEG.

## Introduction

Brain–Computer Interface (BCI) systems provide means by which one could use the brain activity measured either invasively or non-invasively to interact with an external device or their environment^1^. These systems record the brain activity, process the signal, and translate relevant features into commands that can be used to control a virtual or physical machine such as a computer, robot, exoskeleton, prosthetic, or even a digital avatar^2^. BCI systems are currently being used in both assistive modes such as providing means for individuals who are paralyzed to control external devices/communicate or as a rehabilitation tool to promote or improve their recovery process^3^. BCI systems have also proved to be useful in assisting individuals with different neuromuscular and neurological disorders such as spinal cord injury^4^, stroke^5^, cerebral palsy^6^, etc. Thus, BCI systems can compensate, restore or replace their reduced functional capabilities and facilitate neural recovery.

A typical BCI system contains multiple stages of pre and post-processing. The artifact removal stage contains different pre-processing steps which handle most of the artifacts that contaminate the brain signals. This is usually followed by a feature engineering stage wherein the most relevant features for the particular task of interest are identified. These features are then used to train a classifier/regression model to generate the commands for controlling an external device^7^.

Recent advances in machine learning and deep learning-based decoders have led to significant improvement in decoding capabilities using electroencephalography (EEG). Lately, with the advancements in deep learning (DL), studies adopting such models as decoders have exponentially increased. DL models use a computational framework that has multiple layers that learn representations at multiple levels of abstraction. In addition to improving the predictive power, the utility of DL is mainly inspired by the possibility of removing this multi-stage processing of EEG. Many studies have been using deep learning models to function in an end-to-end manner wherein the same model is supposed to handle the artifacts, identify relevant features, as well as perform decoding^8, 9^. Indeed, over 60–70% of studies do not handle artifacts when using deep learning models^8–10^. The possibility of not handcrafting the features required for decoding is also an advantage of using DL models. The model would be able to automatically identify the relevant features thus not limiting the decoding to the hand-picked or pre-selected features. A review by Roy et al.^9^ reported that studies have reported a median decoding increase of 5.4% between DL algorithms and traditional ML algorithms demonstrating the benefit of using DL models as a decoder. However, these models do suffer from poor interpretability and explainability which limits their widespread adoption in spite of the performance improvement, especially in industries such as healthcare^11, 12^. Therefore, there exist concerns on whether this improvement in decoding is from learning the underlying true data distribution or learning spurious artifacts present in the data^13, 14^. Interpreting how and why a model is arriving at specific decisions will be critical to eliminating similar biases in algorithms.

**Figure 1:**
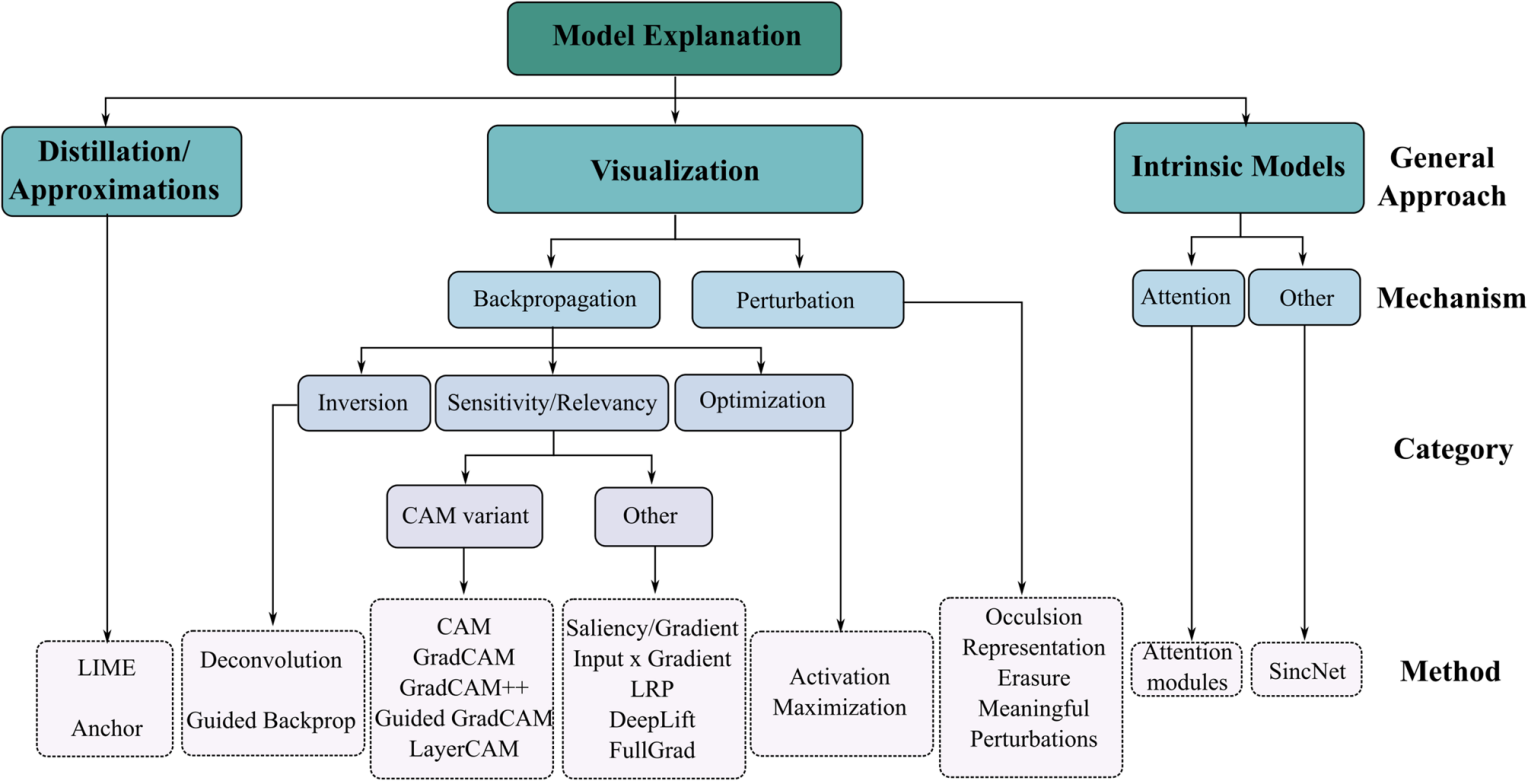
Different types of explanation approaches in neural network models.

Even though there exist many variants of the algorithms being developed to interpret the neural network models, the broad majority of them could be categorized into three categories: Model Distillation, Visualization methods, and Intrinsic methods^15^. A summary of these different types of model explanations is given in Fig. 1.

1. *Distillation/approximations methods:* A group of approaches tries to approximate the DL models with simpler models whose input-output behavior mimics that of the DL model. Later, by interpreting the simpler model, insights into how the complex model works can be obtained. These approaches are broadly labeled under the category of distillation methods. One of the most popular among these methods would be the use of the Local Interpretable Model-agnostic Explanations (LIME) method^16^.
2. *Visualization:* Visualization methods are approaches that in general highlight the most important feature or attribute present in the input that affects the decision of the model through different visualization. One of the most common approaches is the saliency maps which highlight the important segment of the input. These could further be divided into different categories based on how they are implemented. The majority of the approaches developed in this category are based on back-propagation^17^. The gradient/relevancy score for a particular class or neuron is back-propagated in some form for these approaches. The most common and oldest approach is the Gradient approach^18^ which is estimating the gradient of the output with respect to the input. Variants of the simpler models have been developed which are more robust and less noisy like FullGrad^19^, Input X Gradient (IxG), Layerwise Relevance Propagation(LRP)^20^, DeepLift^21^ or different approaches of class activation maps likes GradCAM^22^, GradCAM++^23^, LayerCAM^24^, GuidedGradCAM^25^, ScoreCAM^26^ etc. There are a few methods that attempt to reverse the forward operations (’Inversion’) in a CNN such as Deconvolution^27^ and Guided Backpropagation^28^. Other approaches like activation maximization involve adding an additional ’optimization’ step wherein it tries to create an input that maximizes the score for a particular class/filter of interest^18^. Through all of these methods, the researcher gets additional context through different ways of scientific visualization on what drives a model decision.
3. *Intrinsic methods:* Intrinsic methods involve either developing models which provide an explanation for the decision as part of its model output or those in which explanations can be extracted from the architecture rather straightforwardly way^15^. Some common methods involve models using the attention mechanism^29^. The attention mechanism generates a contextual vector for downstream processing by learning a conditional distribution over the input. Some studies on the other hand engineer the deep network to perform specific meaningful functions which are easily interpretable. One such approach is the development of SincNet^30^ which is based on parameterized sinc functions wherein the model learns cutoff frequencies for the filter banks. This allows for more easily interpretable filters as the most highly activated units would correspond to a particular frequency band.

The needed emphasis on explainability hasn’t picked up a similar pace in popularity compared to deep learning in general for EEG applications. The adoption of explainability for deep learning models in the research involving EEG is still very rare. In the EEG literature, a majority of the model explanations are based on the visualization method using the backpropagation approach. A brief literature review is detailed in the section below. The scope of the paper will therefore be limited to the visualization approach as this is also the most extensively developed explainability method in other domains as well^31, 32^. Limiting the scope to these methods further allows for a more straightforward comparison of their effectiveness. Recent research in computer vision has shown that many of these visualization-based approaches when applied to images have reliability issues^33, 34^. Adebayo et al. showed that visual inspection of model explanations alone can mislead into giving compelling cases. They demonstrated that many of the commonly used explainable methods lack sensitivity to the model and the data generating process^34^. In that study, they randomized the labels and separately reinitialized the model weights. Then they hypothesized that if the model was specific to data and the trained model the explanations should be significantly different with randomization. However, they found that many methods were invariant to these manipulations and only gradients and GradCAM passed their sanity checks. In a separate study Kindermans et al. show that many methods do not satisfy input invariance either^33^. Most of these studies in EEG limit visualization to either one example or an average of one subject. Thus, it is not clear whether the proposed methods would generalize to other datasets. Therefore, it remains unclear which explainability method(s) are robust and reliable when applied to EEG data, and whether or not these methods are sensitive to only certain features in EEG. The sensitivity element is equally important on top of robustness because unlike images, EEG is a bit more complex with features in multiple domains such as temporal, spectral, and spatial domains all equally relevant. Looking at raw time series is less intuitive relative to looking at an image. Also, finding the ground truth in real EEG is a challenging task, particularly with the lower values of signal-to-noise ratios (SNR). Even the same task repeated might have a large source of variability due to the nature of how the human brain works, the influence of the environment, etc. Knowing the exact location of a particular feature in time could be difficult to ascertain when looking at individual trials as well. In addition, often multiple features and noise superimpose making it difficult to know which feature the model is sensitive to.

To address some of these challenges, in this study, we introduce a framework wherein we use simulated EEG to compare different deep-learning explanation methods for EEG applications. The use of simulated data allows the isolation of distinct EEG features. This further allows the production of selective and controlled variations of these features. Here, we test twelve heatmap-based methods on simulated EEG to understand the ground truth sensitivity and robustness of these methods for varying levels of SNR. The sensitivity to detect three fundamental attributes in EEG, specifically the temporal, spectral, and spatial properties are evaluated. We provide a more objective assessment of the robustness and sensitivity of these explainability approaches to these different attributes in EEG. This work compares the strengths and weaknesses of these methods to better understand the pitfalls and provide recommendations for their appropriate application in EEG research.

### Literature review

To better quantify the number of studies that employ explainability approaches when using deep learning on EEG, a literature review was conducted using the Web of Science. The advanced search option was used with the criterion ((AB = (EEG) OR AB = (Electroencephalography)) AND (AB = (neural network) OR AB = (deep learning) OR AB = (CNN) OR AB = (Convolutional Neural Network) OR AB = (Recurrent Neural Network) OR AB = (LSTM) OR AB = (GRU))) AND (ALL = (interpretability) OR ALL = (explainability) OR ALL = (interpretable)). The search conducted in November 2021 gave a total of 65 publications. Among these 30 did not use any specific explainability method in the paper. They either only refer to interpretability/explainability in the paper for discussion purpose or is not relevant. A few of the papers that include interpretability in title/abstract used hand-crafted features to train the model and refer to them as “interpretable models”. These studies were also not included. Two papers were not considered because of poor quality. After removing these papers, only 33 studies remained that used some form of model explanation. On the other hand, studies without the part (ALL = (interpretability) OR ALL = (explainability) OR ALL = (interpretable)) in the advanced search provided a total of 5951 papers suggesting the studies including model explanation currently is less than 0.6%.

**Figure 2:**
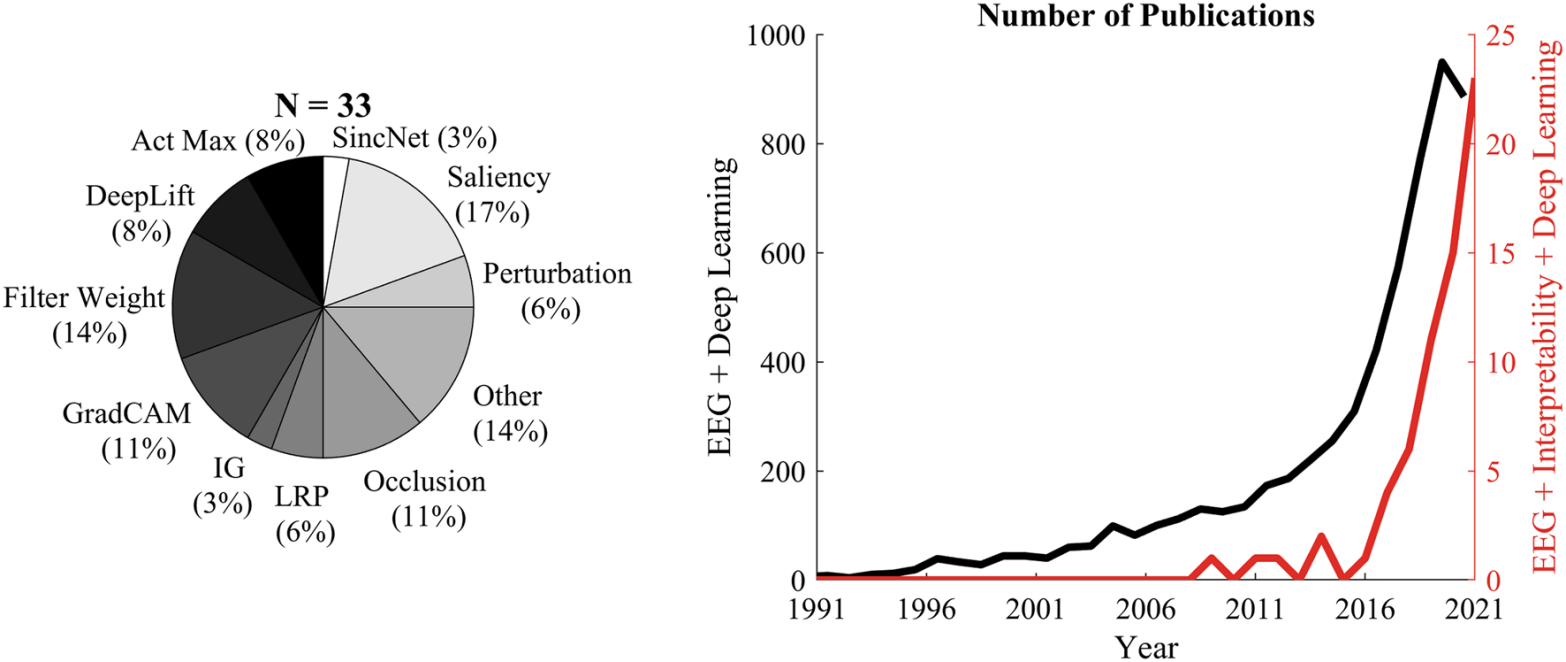
Left: Pie chart showing the distribution of methods used in the screened studies from the web of science search. Right: Trend showing the number of EEG publications using deep learning, with and without explainability (not screened).

The types of methods used in the 33 studies are summarized in Fig.  2. The majority of the studies use some form of heatmap approach. These heatmap approaches highlight the part of the input data the model is looking at to arrive at the correct prediction. The most commonly method (Saliency) is also the most simplest wherein the gradient w.r.t. input was computed^35–42^. The next commonly used method is plotting the convolutional filters directly; usually, the convolutional filters that have a kernel spanning the entire EEG channels (spatial convolutional layer weights)^43–46^. However, looking at the raw weights does not directly indicate whether they are class-specific features or not. Considering there is a large number of filters, the ideal combination of filters that contribute positively to the prediction would be difficult to discern. Also, previous studies have shown that significant non-zero weights can be observed for channels whose activity can be independent of the underlying cortical activity^47^. Many other studies used occlusion-based model explanations wherein they occlude or zero out parts of the input to identify the most sensitive region. However, occlusion methods are not ideal when there are dependencies between non-local features. In that case, it has to be known apriori how to define the mask to include these dependencies (width, the shape of the mask, etc). Other studies have used more complex versions of back-propagation approaches. E.g. Sturn et al. used LayerWise Relevance Propagation (LRP) to identify scalp relevancy associated with motor imagery^48^. Similarly, Lawhern et al. used the Deep Learning Important FeaTures (DeepLift) method^46^ for motor imagery and error-related negativity response task. Ravindran et al. used GradCAM to demonstrate that CNN was learning from common perturbation evoked potentials in single-trial EEG^49^. Ravindran et al. later developed an approach combining clustering and gradcam explanations to demonstrate that these decoders were not biased by artifacts^50^. However, when artifacts were not handled, the model was learning from artifacts. Another approach a good number of studies have used is the activation maximization approach^18^, which synthetically generate inputs that maximally activate a particular neuron, typically the final layer neurons^51–53^. Few studies attempted a perturbation approach in which they perturb the input and evaluate the change in output^54, 55^. The other category includes studies that use approaches not commonly used. Most of them either visualize clustering of hidden layer activation to show class separation^56^ or show a correlation of hidden layer activation to different features^57, 58^.

## Results

The five fold cross-validated decoding accuracy for all the combinations of SNR and conditions is summarized in Table 1. As expected, with lower SNR, the decoding performance decreases. The chance level of spatial, spectral, and temporal conditions are 50%, 25%, and 25% respectively since the classes are balanced. The cross-validated robustness and sensitivity measures were estimated for each of the three conditions for different levels of SNR. The following subsections give the comparison for each of the conditions.

**Table 1:** Cross validated test accuracy for different set of simulated data.

SNR (db)	Test accuracy (%)		
	Spatial	Temporal	Spectral
− 3.5	99.0 ± 0.18	99.54 ± 0.08	98.01 ± 0.5
− 12	94.26 ± 0.35	95.4 ± 0.3	84.5 ± 7.9
− 16	87.8 ± 0.5	90.2 ± 0.3	70.05 ± 8.3
− 19	82.14 ± 0.78	84.9 ± 0.6	57.1 ± 4.6
− 23	71.65 ± 5.2	73.7 ± 0.7	42.0 ± 2.5

### Event related potential component (temporal precision)

**Figure 3:**
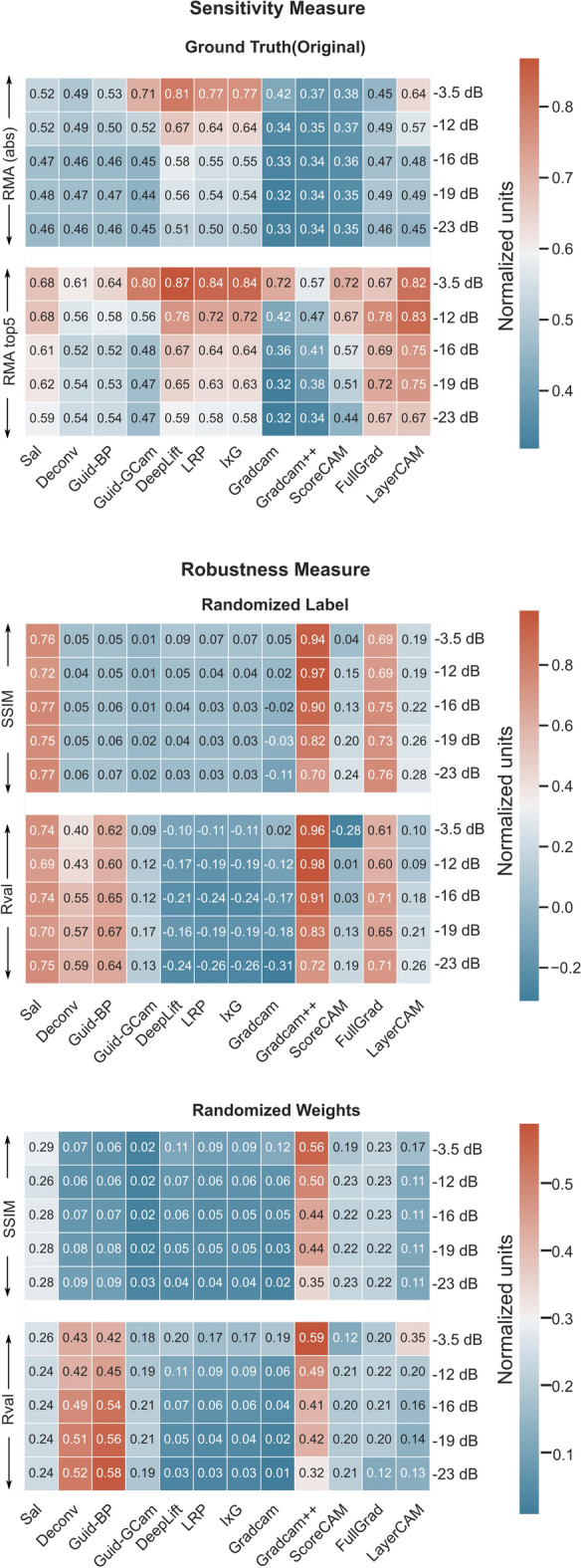
Comparison of the cross-validated metrics for different explanation methods with and without label/model weight randomization for detecting ERP components.

The averaged cross-validated performance metrics are summarized in Fig. 3. From the Relevance Mass Accuracy (RMA) measure, Deeplift was found to be the most accurate/sensitive followed by LRP and I × G to localize the ERP component. This was followed by Guided GradCAM and LayerCAM. On the other hand, GradCAM++ was the worst at temporal precision, followed closely by GradCAM and ScoreCAM.

When the similarity of original explanations was compared to that with randomized labels, it was observed that methods like GradCAM++, Fullgrad, and Saliency have very similar explanations suggesting that their explanations are not class-specific. Similarly, Deconvolution and Guided Backpropagation also yielded a high correlation with the original true explanation. DeepLift, LRP, I × G, and GradCam were the most robust.

In the case of randomized weights, Deconvolution and Guided Backpropagation had the highest R-value followed by GradCAM++. For the Structural Similarity Index (SSIM), GradCAM++ had the highest value followed by Saliency, FullGrad and ScoreCAM. DeepLift, LRP, I × G were still having low values.

Overall, Deeplift was found to be the best closely followed by LRP and I × G,. They had a good trade-off in both robustness and sensitivity whereas GradCAM++ was the worst. Even though Saliency, Guided Backpropagation, and LayerCAM had good sensitivity, they were not very robust to randomizing labels and weights.

### Spectral perturbation (frequency)

**Figure 4:**
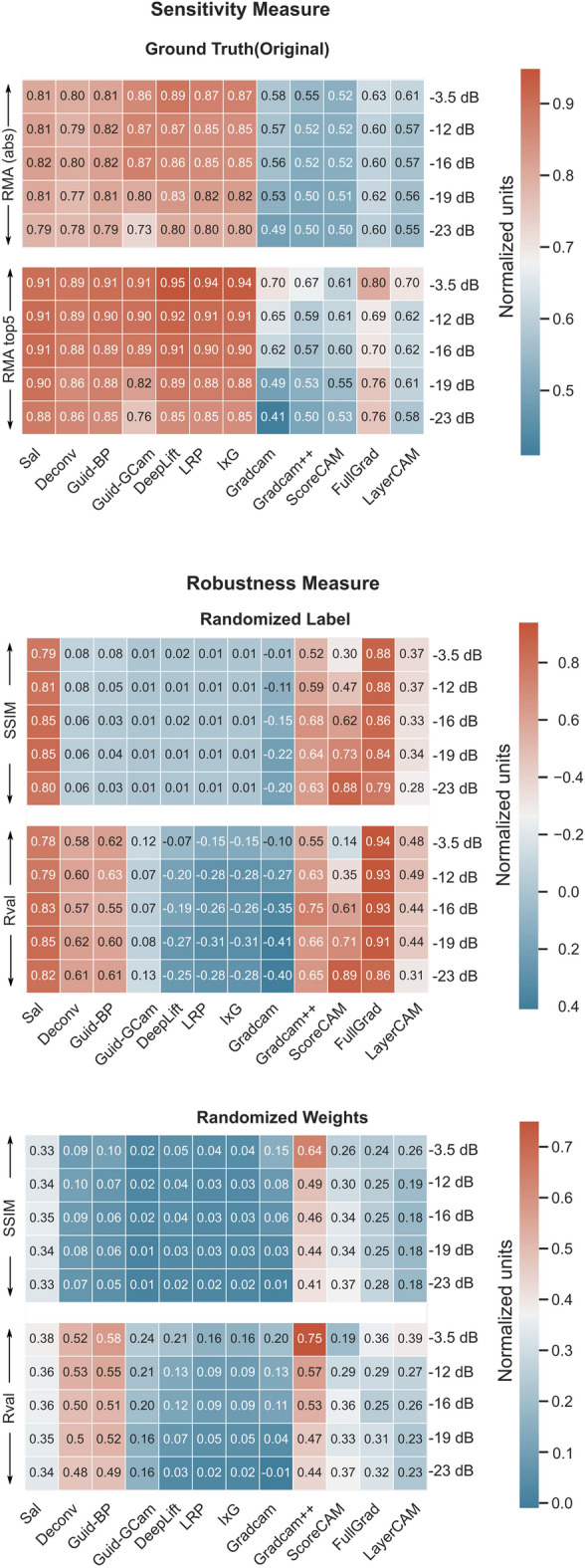
Comparison of the cross-validated metrics for different explanation methods with and without label/model weight randomization for detecting spectral perturbation features.

The averaged cross-validated performance metrics are summarized in Fig. 4. From the RMA measure, most measures do have high accuracy but Deeplift was still the most accurate/sensitive method. This was closely followed by LRP, I × G, Guided G-cam, Guided Backpropagation, Saliency, Deconvolution, and FullGrad. ScoreCam, GradCAM++ was the worst followed by GradCAM and LayerCAM.

When the similarity of original explanations to that with randomized labels is compared, like before, it was observed that GradCAM++, Fullgrad, Saliency, Guided Backpropagation and Deconvolution have very similar explanations suggesting their explanations are not class-specific. Similarly LayerCAM and ScoreCAM also yielded a high correlation with the original true explanation. DeepLift, LRP, I × G, GradCAM, Guided GradCAM were the most robust.

In the case of randomized weights, GradCAM++, Deconvolution, Guided Backpropagation had the highest R-value followed by Saliency, FullGrad, ScoreCam, LayerCAM, and Guided GradCAM. For SSIM, GradCAM++ had the highest value follower by Saliency, FullGrad, and ScoreCam. DeepLift, LRP, I × G, and GradCAM were still having low values.

Overall, Deeplift was found to be the best closely followed by LRP and I × G. They had a good tradeoff in both robustness and sensitivity whereas GradCAM++ was the worst. Even though Saliency, Deconvolution, Guided BP, Guided GradCAM, and FullGrad had good sensitivity they were not very robust to randomizing labels and weights.

### Scalp distribution (spatial)

**Figure 5:**
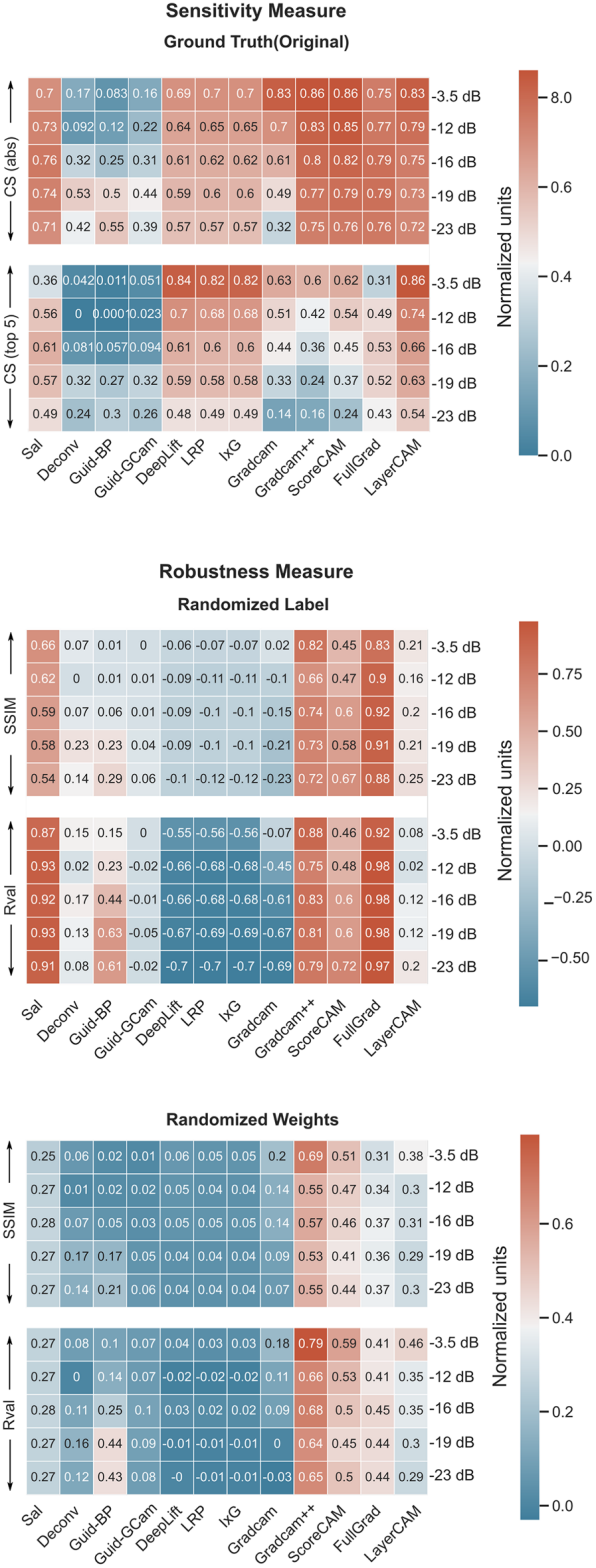
Comparison of the cross-validated metrics for different explanation methods with and without label/model weight randomization for detecting spatial features.

The averaged cross-validated performance metrics are summarized in Fig. 5. Here, cosine similarity was used instead of RMA as there exists a non-zero ground-truth value in all channels due to volume conduction. Here, unlike other measures, based on cosine similarity, it was found that on the true explanation, GradCAM, and ScoreCAM had the highest RMA followed by GradCAM++, FullGrad and LayerCAM. DeepLift, LRP, and I × G still had high values but were lower than the other measures. Guided Backpropagation, Deconvolution, and Guided GradCAM were the worst for spatial relevancy. Even though GradCAM has high sensitivity, its performance drops much fast with SNR lower than 19 dB compared to other methods.

However, when the similarity of original explanations was compared to that with randomized labels, the measures like GradCAM++, ScoreCAM, and Fullgrad which had the highest sensitivity to ground truth, also had the most similarity to the randomized label explanation. Saliency and Guided Backpropagation also had high similarities to the original explanation. DeepLift, LRP, I × G, GradCAM, and Guided GradCAM were the most robust.

Similarly, in the case of randomized weights,GradCAM++, ScoreCAM, and Fullgrad which had the highest sensitivity to ground truth, also had the most similarity to the randomized label explanation. Saliency and Guided Backpropagation also had high similarities to the original explanation. In addition, randomizing weights had a high similarity for LayerCAM as well. DeepLift, LRP, I × G, GradCAM, and Guided GradCAM still remain the most robust.

Overall. GradCAM, Deeplift, LRP, and I × G were the better approach and had a good tradeoff in both robustness and sensitivity. GradCAM++ was the worst. Even though ScoreCAM, FullGrad, LayerCAM, and Saliency had good sensitivity, they were not robust to randomizing labels and weights.

## Discussion

**Figure 6:**
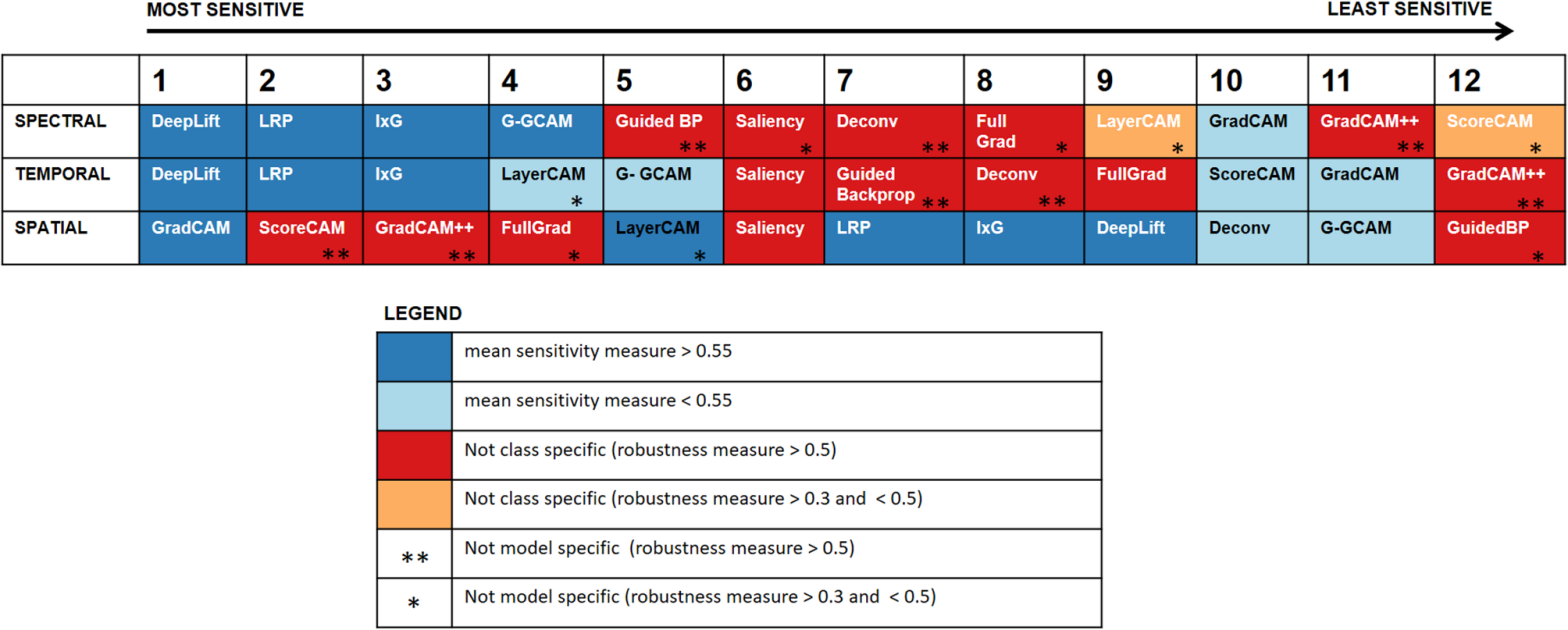
Comparison of the cross-validated metrics for different explanation methods with and without label/model weight randomization for detecting spatial features; LRP: layerwise relevance propagation; IxG: integrated gradient; G-GCAM: guided-GradCAM; Guided BP: guided back-propagation; Deconv: deconvolution.

Including explainability approaches in deep learning is critical to understanding the operation of the model, identifying the most relevant features with discriminative power, and generating scientific insights about the datasets. However, choosing these approaches requires a good understanding of the strengths and weaknesses of the methods available when applied to EEG. Twelve heatmap-based visualization methods were systematically compared for their ability to detect different fundamental attributes of EEG. Using a simulation framework allows us to limit and understand the exact feature from which the model can learn from. Using real EEG, it is very difficult and challenging to ensure the model is only learning from a particular feature and to know the true ground truth available, their location, duration, etc. For the same reason, it would be very difficult to compare the methods on how well they capture the ground truth signal as well. The robustness and accuracy of these methods to varying temporal, spectral, and spatial attributes of EEG for different signal-to-noise ratios were compared. Figure 6 gives a high-level summary of the different comparisons. The methods which have a mean sensitivity measure greater than 0.55 (higher is better) are indicated by the dark blue color. The red color indicates the particular method for the condition being considered is not class-specific (robustness measure > 0.5; higher is worse). Similarly, the orange color indicates the method is not class specific with robustness measure > 0.3 but < 0.5. If the method is not model specific it is indicated by the asterisk “*” symbol. Here, if the robustness measure > 0.5, they are marked with “**” and if the robustness measure > 0.3 and < 0.5, it will be indicated by a single “*”.

Evaluating the robustness and sensitivity measures, even though many measures show high accuracy/sensitivity to the feature of interest, they are not class or model-specific. E.g., Saliency/Gradient is a basic yet one of the most commonly used model explanation methods in EEG^38, 40–42^. They also have high sensitivity to detect spectral perturbation and relevant channels as well. However, randomizing the model weights or labels yielded a very similar explanation to the original one. This suggests that they are not model or label-specific. Therefore, this method should be used with caution. A similar observation was found for many of the methods like Deconvolution, Guided Backpropagation, ScoreCAM, FullGrad, LayerCAM, and GradCAM++ as well. GradCAM++ was one of the least reliable explanation methods.

On the other hand, DeepLift, Input × Gradient, and LRP was found to be both accurate as well as robust in all three cases (spatial, temporal, and spectral). Looking at the explanation metrics, LRP with epsilon rule and Input × Gradient share very significant similarities. This is because previous studies have shown that when all the non-linearities involved are ReLU, epison rule-based LRP approximates to Input × Gradients^59^. There exist multiple studies in Computer Vision that assessed the unreliability of Saliency map-based approaches^34, 60^. However, these studies do not measure the accuracy of these explanation methods. This is an important question because, in the study by Adebayo et al.^34^, they identified that GradCAM was one of the most reliable/robust explanation methods available. In this study, we do show that even though the robustness aspect is preserved in all the 3 conditions, GradCAM is not ideal in the case of spectral perturbation and temporal data conditions. The reasoning for that comes from the framework itself. GradCAM as well as the general class activation maps, compute the model explanation w.r.t. the last convolutional block. With successive pooling and convolution operations, the temporal resolution of the activation in the final convolutional layer would be small. These methods get an estimate of the relevant input by performing a bilinear interpolation to upsample to the input dimension. These will lead to reduced temporal resolution, a key attribute in EEG. However, when we are not interested in the temporal aspect, but instead want to look at spatial relevancy, GradCAM was found to be the most accurate method. Another limitation of using GradCAM which needs to be checked for was that their performance decreased much faster than other methods when the SNR decreased i.e. when the model confidence dropped. One additional point to keep in mind if researchers plan on using GradCAM is that many of the existing EEG architecture uses a spatial convolutional layer in the initial layers. This spatially mixes the information across channels and the succeeding layers do not have channel-independent data. Therefore, using GradCAM in such a case will not be able to produce channel relevancy as the last convolution is purely temporal data. So, this study recommends researchers adopt heatmap-based model explanation methods to either use DeepLift or Layerwise Relevance Propagation in general to explain deep learning studies. However, unless the decoding is poor, GradCAM is still a good alternative for estimating spatial relevancy.

There are several reasons why DeepLift could be performing better than the other methods being tested. Traditional gradient based methods, such as saliency map and gradient backpropagation for instance, have the issue of saturation^61^ when using certain activation functions. In these saturation regions, the gradient becomes zero, effectively masking their effects. Similarly, these methods also have issues when dealing with “dead neurons” which are neurons that have no activity^62^. This is particularly relevant when using ReLU as it zero’s out activity for certain inputs. However, DeepLift avoids this issue as it makes use of a baseline and has the conservation property. It ensures that the sum of the contribution scores for the input would add up to the difference between the model prediction for specific input and that of the baseline. By redistributing the contribution of each neuron’s activation across the input features w.r.t. the baseline, DeepLift overcomes the saturation problem. Hence they are more flexible with different activation functions with saturation or “dead neuron” effects. Also since DeepLift distributes a neuron’s activation across the input features using the conservation property, it might be able to capture the interactions between the input features better. DeepLift also provides layer by layer breakdown unlike methods like GradCAM for instance which is limited to a specific layer of interest. DeepLift considers the contributions of each layer and neuron to the final prediction. This could provide both local and global explanations.

Overall LRP or DeepLift was the most reliable method of all. They were also the most accurate in identifying the ground truth. Even though GradCAM is one of the most robust methods, they fail when the SNR is either low or in the case wherein temporal precision is critical.

These method when added to existing studies will provide additional context to evaluate the bias of the models to spurious correlations or artifacts. There exist multiple ways in which integrating explainable methods could be beneficial when developing BCI. For instance, consider a model for decoding motor imagery for stroke rehabilitation purposes. Ideally, a model should learn neural features from regions of the brain which has representations of limb movement (typically motor-related signals). Say a DL model exhibits significant performance gain compared to traditional models, but if the model is learning from irrelevant noise signals instead of motor-related potentials, the rehabilitation will not be effective and the high-performance increase becomes insignificant. Ideally, the predictive models should utilize neural features associated with the task-specific region to induce neurorecovery, rather than an unrelated neural activity that is not associated with the motor task. Similarly, if explanations could be provided on what the model was looking for when making the decisions, the researchers/end users who are hesitant to use these models could be more open to their adoption.

One of the advantages of using DL is the possibility it offers to avoid the need for hand crafting features- so-called feature engineering. The model can automatically identify relevant patterns required for decoding. This is another important area in which explainability would offer tremendous possibilities. Understanding what the models are looking at could lead to new scientific discoveries and progress the field forward. This can also be useful for in making useful implementation decisions. For example, in a recent study Ravindran et al.^50^ demonstrated the balance perturbations could be detected from single trial EEG. From the model explanations, we can identify the subset of channels which were deemed most important. When developing a wearable system for fall prevention, we could then use this information to select a smaller montage thus reducing the set-up time, cost, etc. for the system. Similar decision making could be possible for decoders developed for different BCI tasks.

Additionally, using explainability approaches allows us to understand the failure modes in the model, giving valuable insights about the model. These methods will help debug the model by identifying some of its limitations and mistakes thereby improving the model. Overall, there exists multitude of ways in which explainability approaches could be directly useful in improving the BCI.

Some of the limitations and future directions of the analysis are discussed below:

### Approximation error

Synthetic EEG is only an approximation to measured EEG. Many physiological and non-physiological signals and artifacts, which are generally present in measured EEG, are not contained in the synthetic EEG. This can be both an advantage and also a limitation of the simulation approach. There is a possibility of missing some key EEG properties while modeling using simulation. However, in this study, objectivity was prioritized higher to compare the different methods. Moreover, EEG data is quasi-stationary, context-dependent, and influenced by learning. Thus, interpretability models must also account for these factors if they are part of the experimental design. In future studies, with the developed framework, identified confounds and complex modeling could be investigated.

#### High level explanations

The scope of this research is limited to visualization methods that highlight key segments of the input data. However, assessing which specific feature in EEG caused the correct prediction would still be difficult to ascertain. However, combining the methods can help develop insights. Knowing the scalp relevance heatmap can help isolate the relevant channels. Later, checking the relevancy of temporal data can get specificity for temporal localization. Following this with activation maximization^53^ on these channels or other feature perturbation approaches^54^ can give insight into the relevant frequency bands or feature that is being perturbed. This can be followed up with traditional signal processing methods focused on the relevant regions to gain additional insights. This method can identify which features are not sensitive (if any) as well as the regions that are not important and those that can be avoided.

#### Other approaches

Although this research limited the analysis to visualization-based approaches, there are other types of model explanations as summarized in the introduction. Some of these methods could provide better insights. However, exploring all of these iterations is outside of the scope of the study and will be explored in future studies.

## Conclusion and future directions

The approach used here will serve as a benchmark for future researchers to get familiarized with the robustness and effectiveness of multiple explainable techniques; specifically, different heatmap-based attribution methods. The research provides a summary and recommendations to understand when some of these methods fail and what they can capture in EEG. This study is limited to features that are commonly reported in the tasks studied in this research. There could be many other features to test for and the set is not exhaustive. In this study, we kept the scope to a generic CNN architecture particularly since many of the methods such as deconvolution or guided backpropagation, for instance, are only applicable to CNN architectures. This was also informed by the literature which suggests that majority of EEG studies are using CNN models^8^. However, there exists different variants of deep learning models such as CNN with attention modules and/or residual connections, Transformer models, Recurrent Neural Networks etc. This would involve further detailed analysis exploring the effects of these variations. We hope this work will stimulate and introduce a framework wherein future studies can leverage the approach of simulation and use learning from this study to answer some of these interesting questions. Future studies should expand to such model variants and improve upon the framework we introduce here.

Overall, this research identified that some of the most used model explanation methods such as Saliency/Gradient are not class or model-specific. It was found that DeepLift was consistently accurate as well as robust to detect the three key attributes tested here. GradCAM even though was consistently robust, does not have good temporal precision. However, it is still good for detecting spatial patterns for signals with high SNR. Overall, specific recommendations and best practices for the use of back-propagation-based visualization methods for EEG-based decoder design are provided.

## Methods

### Convolutional neural network

**Figure 7:**
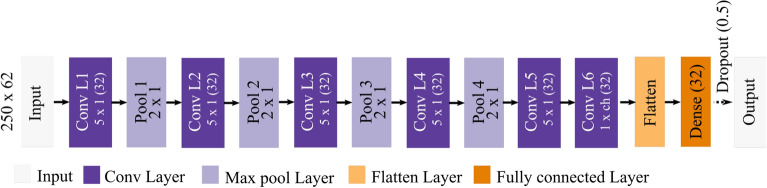
Model architecture: Each block corresponds to different types of layers in the model. The dotted line illustrates the dropout operation during the training phase aimed at reducing overfit. During inference, all units were retained.

The architecture for the model is summarized in Fig. 7. The intention was to use a very generic CNN model without any specialized architectural changes. This was done to ensure generalizability to existing studies. The input to the model is the 1 s EEG window (batch size × 250 samples × 62 channels). Two channels were removed as they are not contained in the forward model. The model consisted of 5 temporal convolution layers of 32 units each (5 × 1 kernel size with a stride length of 1) and 1 spatial convolution layer of 32 units (1 × 62 kernel size). The number of convolutional layers was kept as 6 as the majority of the prior studies used 6 or lower convolutional layers^8^. The filter size was selected such that the total receptive field for the final convolutional block would span at least half the sampling rate (125 Hz). A temporal pooling layer of 2 × 1 pooling dimension with a stride length of 2 was also used after every convolutional filter layer except the last two blocks. The output from these convolutional layers was flattened and fed into a dense, fully connected layer of 32 hidden units followed by an output layer with softmax activation.

A dropout layer with alpha = 0.5 was added in between the dense layer and the output layer to reduce overfitting. Except for the output layer, the model utilized ReLU as the activation function. ReLU was used as the activation function as this was also the most popular activation function used (70% of studies^8^). The proposed model was implemented in python 3.7 using Pytorch library^63^. For each of the conditions (temporal, spatial, and spectral), an independent model was trained to classify the distinct classes. A five fold cross-validation was performed and model explanations and the comparison metrics were estimated on the test set from each fold. The value across the folds are then compared between the type of model explanations.

### Simulated data

**Figure 8:**
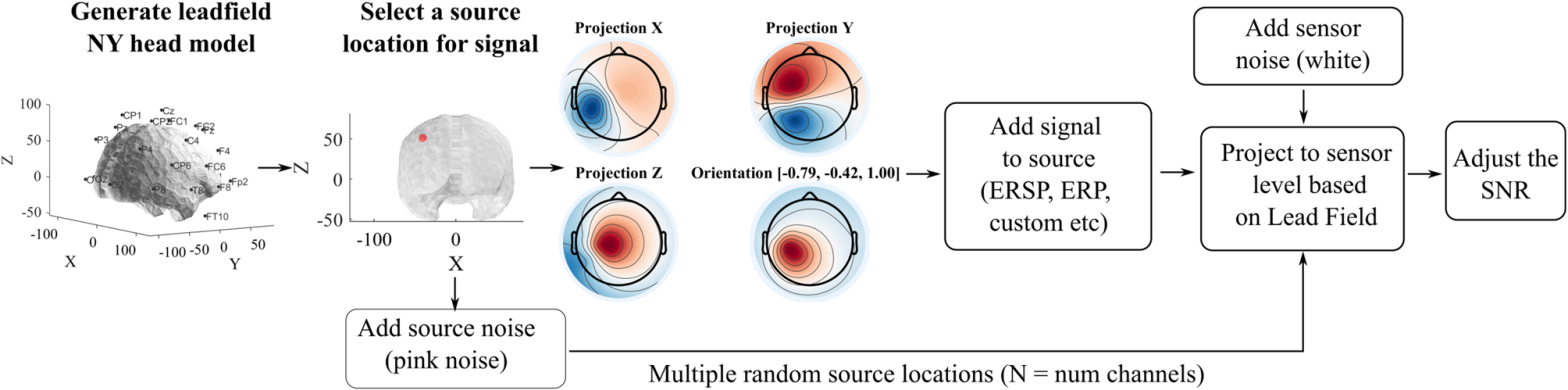
Steps present in generating different types of features in simulated EEG using the SEREEGA toolbox; ERSP: event-related spectral perturbation; ERP: event-related potential; NY: New York; SNR: signal-to-noise-ratio.

To compare the relative performance of different model explanation methods, the SEREEGA library^64^ was used to simulate ground truth EEG features. The typical workflow used to simulate EEG activity using SEREEGA is summarized in Fig. 8. The process starts by defining the lead field matrix and the head model. The New York head model was used for generating the lead field matrix^65^. The toolbox supports the pre-generated leadfield that includes 75,000 source locations which could be projected to 228 sensor locations on the scalp. The New York head model does detailed segmentation of six types of tissues (scalp, skull, cerebrospinal fluid, gray matter, white matter, and air cavities). Later, the source location was selected to project the feature from. The source location could either be randomly selected or chosen manually based on the Montreal Neurological Institute (MNI) coordinates^66^. Later, the orientation for the dipoles was chosen. Each source has a default orientation associated with it. But, the orientation that is either tangential or perpendicular to the scalp for each of the dipoles can also be chosen. For this study, all dipoles are chosen to be perpendicular to the scalp surface to improve the localization of the scalp projection for ground truth.

Once the source and the orientation are selected, an activation/signal would be added to these sources. SEREEGA offers systematic deflections in the time domain to simulate event-related potentials as well as systematic modulations of oscillatory activity to simulate event-related spectral perturbation. The toolbox also allows the simulation of different types of additive noises (pink, white, brown, etc). Once the appropriate signal and noise are added, it allows mixing of the signal and noise in varying proportions such that different combinations of Signal-to-Noise Ratio (SNR) could be achieved at the projected scalp EEG. In addition, uncorrelated white noise was added to simulate sensor noise. Using the combination of signal, noise, source location, and orientation the toolbox allows the creation of ground truth simulated EEG with varying localization capabilities in temporal, spatial, and spectral domains.

**Figure 9:**
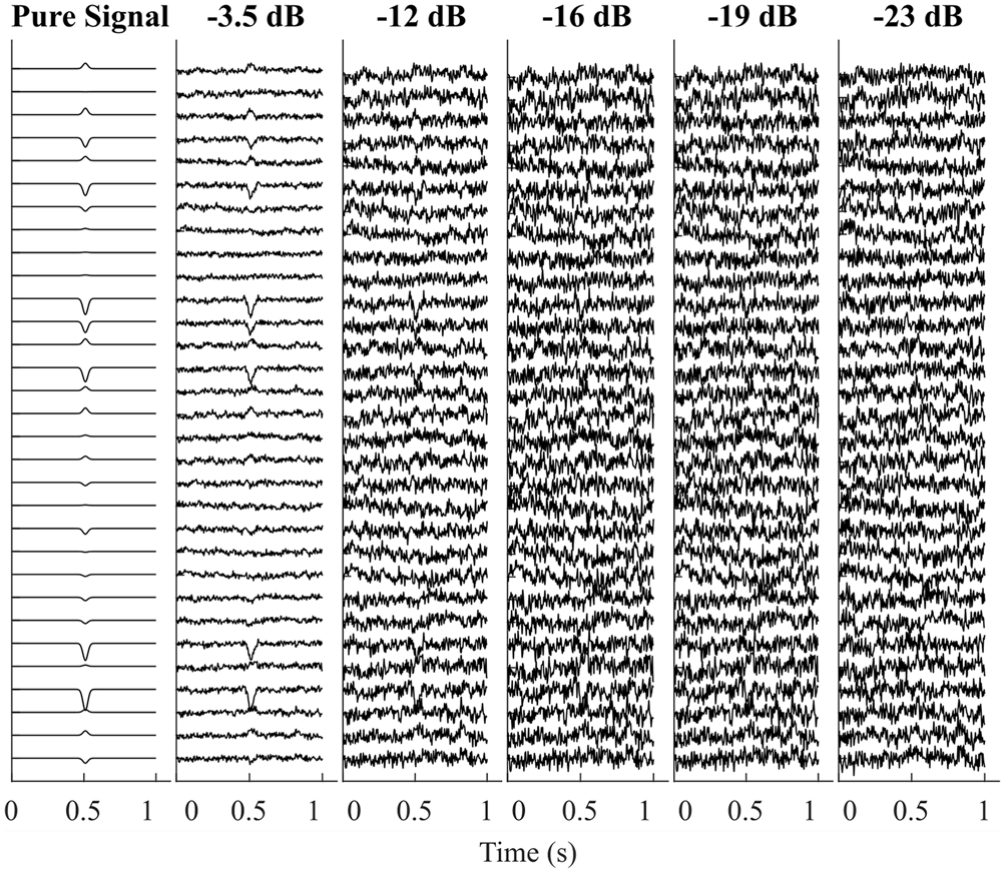
Representative example to demonstrate the effect of varying SNR on an ERP component.

For all the simulations, the leadfield matrix projected onto actiCAP64 channel configuration from the sources was used. The sampling rate was set to 250 Hz with the window size of each simulated epoch 1 s long. To replicate brain noise, sources equaling the number of channels—the number of signal dipoles were uniformly selected randomly across the brain surface and a 5 μV pink noise was added to these sources similar to the simulation replication done by Krol et al.^64^. For each condition, to evaluate the performance impact under varying SNR, the noise was added to yield the following SNR: − 3.5 dB, − 12 dB, − 16 dB, − 19 dB, and − 23 dB. Figure 9 shows an example of the difference when the simulated ERP component gets added with noise at varying SNR.

#### Event-related potential components

**Figure 10:**
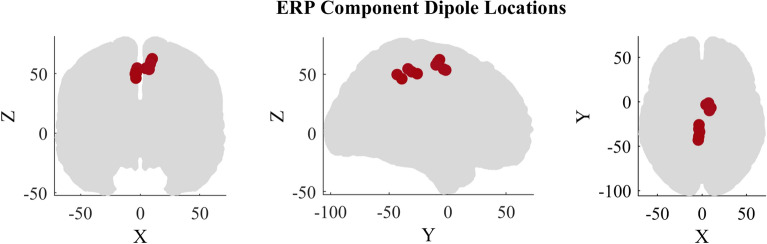
Dipole locations in MNI coordinates for the ERP components.

To evaluate how different model explanations are fair in localizing the temporal aspect of EEG, different ERP components were simulated. Four distinct classes of ERP components were simulated with N = 10,000 per class. For each epoch, the source location was sampled from one among 10 source locations in Table 2. Even though the precise location is not very important, in order to have some constraint, source locations were selected corresponding to perturbation evoked potentials based on ranges suggested in the source analysis results from prior studies associated with perturbation evoked responses (PEP)^67–69^. The source locations in the MNI coordinates are shown in Fig. 10.

**Table 2:** MNI coordinates of the ERP sources.

No.	Dipole location	x	y	z
1	Paracentral lobule	− 9.1	− 8.5	60.2
2	Paracentral lobule	10.1	− 6.9	62.3
3	Paracentral lobule	4.6	− 3.4	54.3
4	Paracentral lobule	8.4	− 9.9	57.9
5	Posterior cingulate	7.5	− 1.6	53.5
6	Precuneus	− 2.6	− 33.9	54.5
7	Posterior cingulate	− 3.5	− 30.7	52.1
8	Precuneus	− 4.1	− 43.2	49.7
9	Isthmus cingulate	− 3.6	− 39.2	46.1
10	Posterior cingulate	− 3.3	− 26	50.4

The following attributes for the source components were tested in the simulation.

1. Class 1: Time locked positive deflection of EEG. Class 1 contained a positive component centered at 60 ± 8 ms latency with a peak width of 50 ± 2 ms. The amplitude of the component was randomly sampled between 1 and 13 μV uniformly. The component’s magnitude and width closely resemble the characteristic range of the P1 component in perturbation-evoked potentials^70^. One among the first 5 source locations from Table 2 was selected randomly as the source location.
2. Class 2: Same properties as Class 1 but different latency (latency difference). Class 2 contained a positive component centered at 900 ± 5 ms latency with a peak width of 100 ± 4 ms. The amplitude of the component was the same as that of Class 1. However, latencies were shifted to avoid overlap between the two classes to better quantify and compare the explainability techniques. One among the first 5 source location from Table 2 was selected randomly as the source location.
3. Class 3: Same magnitude as Class 1 and 2 but negative deflection instead of positive (sign difference). Class 3 consisted of an ERP component with the same amplitude as class 2 but inverted with a latency centered at 500 ± 8 ms and a width of 100 ± 4 ms One among the first 5 source locations from Table 2 was selected randomly as the source location.
4. Class 4: Same magnitude and sign as class 3 but a different source location (source difference). Class 4 consisted of a signal of the same properties as Class 3 except that the source location is different. One among the source location (6–10) from Table 2 was selected randomly as the source location.

#### Spectral perturbations

**Figure 11:**
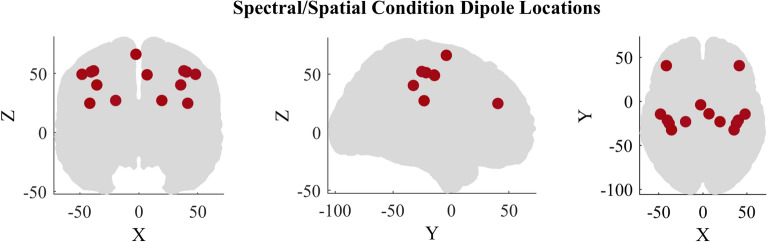
Dipole locations in MNI coordinates for both the spectral and spatial conditions.

To test the sensitivity to detect spectral perturbation events, four separate classes of data were simulated each belonging to spectral perturbation events happening in four separate frequency bands. The magnitude of the signal was set to 0.5–3 μV^64^. For each epoch, the magnitude and the latency were kept the same for all classes and they only differed in their spectral content/frequency. The latency of the center of the spectral burst for each epoch was uniformly random sampled to be between 200 and 500 ms to add a source of variability. The burst width was randomly sampled to be between 400 and 600 ms. The MNI coordinates used for the sources are summarized in Table 3. The source location was referenced based on dipoles associated with motor imagery/execution from prior literature^71–73^. For each epoch, one of the dipole locations was selected at random to act as the source. All the dipole locations are shown in Fig. 11.

1. Class 1: Spectral perturbation in the frequency band of 3–8 Hz. The magnitude, latency, and width of the burst were randomized between epochs.
2. Class 2: Spectral perturbation in the frequency band of 8–13 Hz. The magnitude, latency, and width of the burst were randomized between epochs.
3. Class 3: Spectral perturbation in the frequency band of 14–30 Hz. The magnitude, latency, and width of the burst were randomized between epochs.
4. Class 4: Spectral perturbation in the frequency band of 30–58 Hz. The magnitude, latency, and width of the burst were randomized between epochs.

**Table 3:** MNI coordinates of the dipoles selected for the spectral perturbation and spatial condition simulations.

No.	Dipole location	x	y	z
1	L Superioparietal	− 40	− 21	51
2	R Postcentral gyrus	40	− 21	51
3	L Superioparietal	− 38	− 26	53
4	R Postcentral gyrus	38	− 26	53
5	L Postcentral gyrus	− 48	− 15	50
6	R PostCentral gyrus	48	− 15	50
7	L Cingulate gyrus	− 24	− 24	32
8	R Cingulate gyrus	24	− 24	32
9	L Supramarginal gyrus	− 34	− 32	38
10	R Superior parietal	34	− 32	38
11	L Rostral middle frontal gyrus	− 42	40	25
12	R Caudal middle frontal	42	40	25
13	L Paracentral	0	− 4	65
14	R Posterior cingulate	8	− 12	52

**Figure 12:**
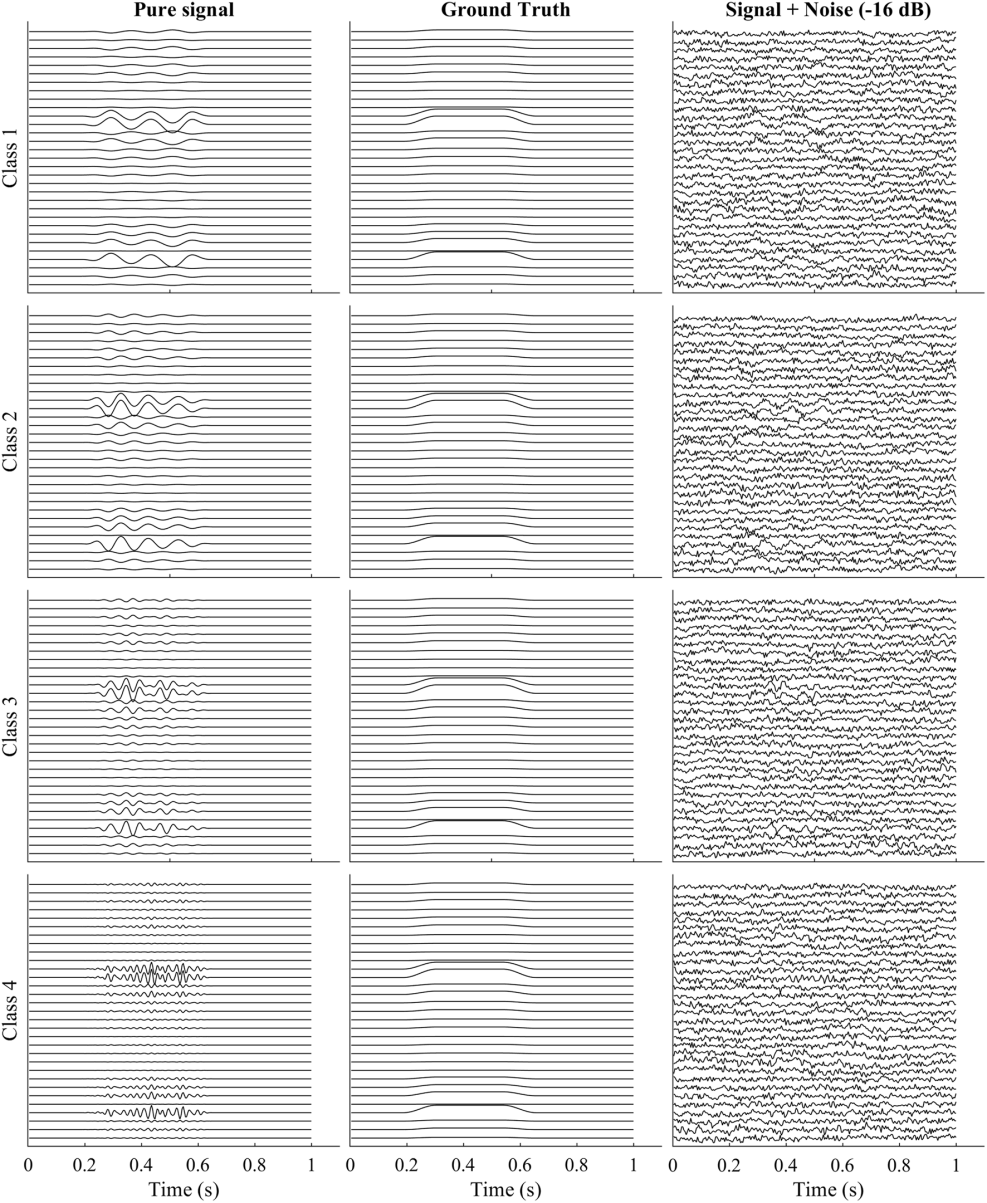
Representative example to demonstrate the effect of varying SNR on a spectral component.

The representative example of simulated EEG from each of the classes is shown in Fig. 12.

#### Spatial precision

Different ERP components and spectral perturbations with identical properties but different dipole location was simulated to assess the channel specificity. The only separation between the two classes created here is the location of the source signal. Class 1 had dipoles localized in the left hemisphere and Class 2 contains dipoles in the right hemisphere. Here the model is expected to learn all the distinct features and localize the correct scalp projection. The dipole source location for Class 1 was randomly selected from all source locations in the left hemisphere in Table 3. Class 2 on the other hand corresponds to locations in the right hemisphere in Table 3

### Robustness and sensitivity analysis

For each condition, the simulated EEG with the respective properties are generated as discussed before. This signal is then forward projected. Noise is later added with varying levels of signal-to-noise ratios as discussed before. To get the ground truth explanation, the tapered window corresponding to the signal location was forward projected using the same lead field matrix. The segment outside of the projected signal would have a value of 0. The section with the signal (across all the channels) was normalized by dividing by the maximum value. The sensitivity/accuracy of each method was compared by evaluating the performance metrics (discussed below) w.r.t. this ground truth data.

To test the robustness of each of the explanation methods, the approach used in Adebayo et al.^34^ was adopted. Once the original explanation was obtained, the explanation after independently randomizing the labels and the model weights was re-computed. This tests whether the explanations are class or model-specific. The similarity of explanations w.r.t. the original explanation based on the absolute Pearson’s correlation coefficient and the SSIM measure (detailed later) was estimated. Ideally, if the model is accurate, it should have high similarity to the ground truth. On the other hand, if explanations are model and/or class-specific, the randomization performed should yield very dissimilar explanations to the original explanations. If the explanations are very similar even after randomizing, it indicates that the explanation is not very robust. The process was repeated for each type of signal/condition and SNR levels for all the explanation methods being compared.

### Explanation methods

The different types of visualization-based explanation methods being compared in this study are detailed below. All the methods were implemented in Python using Pytorch 1.7.0 framework^63^ using either Captum 0.4.0^74^ or the Pytorch-grad-cam toolbox^75^.

#### Gradient/saliency (S)

Gradient or basic Saliency map (Sal) as referred to in some studies is probably one of the earliest yet commonly used model explanation approaches. The gradient gives a measure of how a change in input *x* would change the prediction *S*(*x*) in a small neighborhood around the input^18^. It is given by

1 $$\begin{aligned} Saliency/Gradient = \frac{\partial S}{\partial x}. \end{aligned}$$

#### Deconvolution

Deconvolutions (Deconv) can be thought of as reversing the process done in a convolutional neural network^27^. Essentially attempting to recreate the input from the output activation by running the CNN in reverse top-down. The convolutions get replaced with deconvolutions also called transposed convolution. The filter values are copied after transposing their values. The process also replaces max-pooling layers with unpooling operations wherein the feature map is upsampled depending on the pooling parameters while retaining the maximum value. This is done by storing the position of the maximum value in the forward operation of the CNN. The process is repeated from the layer whose filter is to be visualized back to the input space.

#### Guided backpropagation

Guided backpropagation^28^ (Guided-BP) builds upon deconvolution. It combines vanilla backpropagation at ReLUs (knowing which elements are positive in the previous feature map) with DeconvNets (keeping only positive gradients).

#### Input × gradient

Input × Gradient is another type of attribution method wherein, the gradient was multiplied with the input *x*^76^. The equation to compute the Input × Gradient is

2 $$\begin{aligned} Input \times Gradient = \frac{\partial S}{\partial x} \cdot x. \end{aligned}$$

#### GradCAM

GradCAM is a generalization for Class Activation Map (CAM) as CAM limits the CNN to require a global average pooling layer at the end of the convolutional blocks^22^. GradCAM on the other hand does not require this.

For the $k{th}$ feature map activation $A_k$ in the final convolutional layer of a CNN, the gradient of the score $y_c$ for the class *c* of interest is initially computed. The average score of the gradient w.r.t. each node in the feature map is computed to get an importance value $\alpha _{k,c}$ for the particular feature map. The equation to estimate $\alpha _{k,c}$ is

3 $$\begin{aligned} \alpha _{k,c} = \frac{1}{m \cdot n}\sum _{i=1}^{m}\sum _{j=1}^{n}\frac{\partial {y_c}}{\partial {A_{k,i,j}}}. \end{aligned}$$

Here, $A_{k,i,j}$ is a single neuron/node at position (*i*, *j*) in the feature map $A_k$ of dimension m x n. GradCAM then linearly combines the importance score for each of the feature map and pass them through a ReLU the total relevance score map equals to

4 $$\begin{aligned} GradCAM = \textrm{ReLU} \left (\sum _k^K \alpha _{k,c}A_k \right). \end{aligned}$$

The relevancy score is then upsampled using bi-linear interpolation to the same dimension as the input.

#### GradCAM++

GradCAM++ can be considered as a generalized formulation for GradCAM^23^. This method uses the second and third-order derivatives on the gradients to obtain the gradient weights

#### Guided GradCAM

Guided GradCAM is a combination of GradCAM and Guided Backpropagation to obtain pixel-level granular GradCAM representation^25^. GradCAM is combined with Guided Backpropagation by performing an element-wise product of the two to obtain Guided GradCAM.

#### Layer wise relevance propagation

Layer-wise Relevance Propagation (LRP) redistributes the prediction score for a particular class of interest through a custom backward pass through the model back to the input following a conservation principle^20^.

#### DeepLift

DeepLift is similar to LRP in the sense that it decomposes the output prediction for a particular input by backpropagating the contribution of all neurons in the model to each feature of the input^21^. DeepLift gives a measure of the change in output from a “reference” output w.r.t. the change in input from a ’reference’ input. The reference is a neural input that is task-irrelevant. Here an array of zeros is used with the same dimension as the input^46^.

#### ScoreCAM

ScoreCAM is a perturbation-based expansion to the class activation map framework^26^. ScoreCAM basically tries to mask part of the input and observe the change in prediction score for the class of interest similar to the occlusion approach. However, unlike occlusion, here the mask is obtained by initially forward passing to get the feature map activation. To perturb the input these are up-sampled to the input dimension and smoothed by normalizing to have a value between 0 and 1. Later they are masked based on the activation scores and the masked input is fed into the CNN to compute the prediction score which serves as a weight for the feature map. This process is repeated for all the filters present in the final convolutional layer and pooled to obtain the final ScoreCAM representation.

#### FullGrad

FullGrad is an attribution method that aggregates the gradient for the entire network by decomposing the prediction score into input sensitivity and per-neuron sensitivity components. FullGrad computes the gradient of the biases from the entire network and sums them^19^.

#### LayerCAM

LayerCAM builds on top of GradCAM wherein the class activation maps are extracted for all layers instead of the final convolutional layer as is done in CAM/GradCAM^24^.

### Metrics

The visualization approach assigns relevancy or importance scores to each pixel/data point in the input. To compare different explanation methods, metrics to quantify the similarity of the explanations after randomization as well as, the efficiency in capturing the true underlying ground truth is equally important. For the robustness measure, both the Pearson’s correlation and Structural Similarity index (SSIM)^77^ were used to compare explanations before and after randomization. The output of the visualization methods being compared here can be considered as images with relevancy scores on a pixel basis. SSIM has been demonstrated to have good agreement with human observers when using reference images by quantifying the perceptual difference and has been shown to perform better compared to both mean squared error, as well as the peak signal-to-noise ratio. In addition, the correlation coefficient further quantifies the linear relationship between the two. Ideally, for a robust method, the original explanations should become uncorrelated or minimally correlated w.r.t. the explanation after randomizing.

#### Robustness metrics

The measures used to compare the similarities between the explanations are adapted from Adebayo et al.^34^.

1. Pearson’s Correlation Coefficient: Compute the sample correlation between the explanations yielding a measure of the strength and direction of the linear relationship between the two variables. Here the explanations would initially be flattened out. The equation to compute Pearson’s Correlation Coefficient is
  5 $$\begin{aligned} r = \frac{cov(x,y)}{\sqrt{var(x)} \cdot \sqrt{var(y)}}. \end{aligned}$$
2. Structural Similarity Index (SSIM): Measure the perceptual similarities between two images SSIM. Given two images/inputs, SSIM provides a measure of distortion along the luminance, contrast, and correlation dimensions^78^.

here, *cov*(*x*, *y*) is the covariance between *x* and *y* and *var* corresponds to the variance

#### Sensitivity metrics

To compare the effectiveness of these models in identifying the true signal of interest, two measures to quantify the sensitivity are used. The main goal of evaluating these measures is to ensure that a majority of the top relevancy scores assigned fall in the ground truth region of the data. A ground truth mask is a binary array with a value of one assigned to all non-zero data points in the ground truth and a value of zero for others. The relevance mass accuracy measure quantifies how much of the total relevancy assigned by the methods is localized in the ground truth region. This gives a measure of accuracy.

1. Relevance Mass Accuracy (RMA): Ratio of the total relevancy inside the ground truth mask divided by the sum of the total relevancy assigned for the input^79^. The equation to compute RMA is
  6 $$\begin{aligned} Relevance Mass Accuracy = \frac{R_{within}}{R_{total}}. \end{aligned}$$
  here, $R_{within}$ is the relevancy score assigned by each of the method that falls within the ground truth whereas $R_{total}$ is the total relevancy score assigned by the method. Since in the simulation, the source signal has been assigned to a dipole that projects onto the surface, a non-zero ground-truth value is assigned to all channels due to volume conduction. Therefore, to compare the similarity with the ground truth topoplot representation, a different distance measure of similarity is used for spatial data
2. Cosine Similarity (For Spatial Sensitivity): Cosine similarity computes the cosine of the angle between two non-zero vectors which is equivalent to the inner product of the vectors after normalizing to get unit length^80^. The equation to compute cosine similarity is
  7 $$\begin{aligned} Cosine Similarity = \frac{A \cdot B}{\sqrt{\Sigma {A}} \cdot \sqrt{\Sigma {B}}}. \end{aligned}$$

## Data Availability

The datasets used and/or analyzed during the current study are available from the corresponding author on reasonable request.

## References

[1] Shanechi MM (2019). Brain–machine interfaces from motor to mood. Nat. Neurosci..

[2] Shih, J. J., Krusienski, D. J. & Wolpaw, J. R. Brain-computer interfaces in medicine. In *Mayo Clinic Proceedings*, Vol. 87, 268–279 (Elsevier, 2012). 10.1016/j.mayocp.2011.12.008 PMC3497935 22325364

[3] Chaudhary U, Birbaumer N, Ramos-Murguialday A (2016). Brain–computer interfaces for communication and rehabilitation. Nat. Rev. Neurol..

[4] Salisbury DB, Parsons TD, Monden KR, Trost Z, Driver SJ (2016). Brain-computer interface for individuals after spinal cord injury. Rehabil. Psychol..

[5] López-Larraz E, Sarasola-Sanz A, Irastorza-Landa N, Birbaumer N, Ramos-Murguialday A (2018). Brain-machine interfaces for rehabilitation in stroke: A review. NeuroRehabilitation.

[6] Ponce, P., Molina, A., Balderas, D. C. & Grammatikou, D. Brain computer interfaces for cerebral palsy. Cerebral Palsy-Challenges for the Future (2014).

[7] Paek AY (2021). A roadmap towards standards for neurally controlled end effectors. IEEE Open J. Eng. Med. Biol..

[8] Craik A, He Y, Contreras-Vidal JL (2019). Deep learning for electroencephalogram (EEG) classification tasks: a review. J. Neural Eng..

[9] Roy Y (2019). Deep learning-based electroencephalography analysis: A systematic review. J. Neural Eng..

[10] Al-Saegh A, Dawwd SA, Abdul-Jabbar JM (2021). Deep learning for motor imagery EEG-based classification: A review. Biomed. Signal Process. Control.

[11] Samek, W. & Müller, K.-R. Towards explainable artificial intelligence. In *Explainable AI: Interpreting, Explaining and Visualizing Deep Learning*, 5–22. (Springer, 2019).

[12] Samek, W., Wiegand, T. & Müller, K.-R. Explainable artificial intelligence: Understanding, visualizing and interpreting deep learning models. arXiv preprint arXiv:1708.08296 (2017).

[13] Lapuschkin S (2019). Unmasking clever Hans predictors and assessing what machines really learn. Nat. Commun..

[14] Buckner C (2020). Understanding adversarial examples requires a theory of artefacts for deep learning. Nat. Mach. Intell..

[15] Xie, N., Ras, G., van Gerven, M. & Doran, D. Explainable deep learning: A field guide for the uninitiated. arXiv preprint arXiv:2004.14545 (2020).

[16] Ribeiro, M. T., Singh, S. & Guestrin, C. “Why should i trust you?” Explaining the predictions of any classifier. In *Proceedings of the 22nd ACM SIGKDD International Conference on Knowledge Discovery and Data Mining*, 1135–1144 (2016).

[17] Hecht-Nielsen, R. Theory of the backpropagation neural network. In *Neural Networks for Perception*, 65–93 (Elsevier, 1992).

[18] Erhan D, Bengio Y, Courville A, Vincent P (2009). Visualizing higher-layer features of a deep network. Univ. Montreal.

[19] Srinivas, S. & Fleuret, F. Full-gradient representation for neural network visualization. arXiv preprint arXiv:1905.00780 (2019).

[20] Bach S (2015). On pixel-wise explanations for non-linear classifier decisions by layer-wise relevance propagation. PLoS ONE.

[21] Shrikumar, A., Greenside, P. & Kundaje, A. Learning important features through propagating activation differences. In *International Conference on Machine Learning*, 3145–3153 (PMLR, 2017).

[22] Selvaraju, R. R. *et al.* Grad-cam: Visual explanations from deep networks via gradient-based localization. In *Proceedings of the IEEE International Conference on Computer Vision*, 618–626 (2017).

[23] Chattopadhay, A., Sarkar, A., Howlader, P. & Balasubramanian, V. N. Grad-cam++: Generalized gradient-based visual explanations for deep convolutional networks. In *2018 IEEE Winter Conference on Applications of Computer Vision (WACV)*, 839–847 (IEEE, 2018).

[24] Jiang P-T, Zhang C-B, Hou Q, Cheng M-M, Wei Y (2021). Layercam: Exploring hierarchical class activation maps for localization. IEEE Trans. Image Process..

[25] Selvaraju, R. R. *et al.* Grad-cam: Why did you say that? arXiv preprint arXiv:1611.07450 (2016).

[26] Wang, H. *et al.* Score-cam: Score-weighted visual explanations for convolutional neural networks. In *Proceedings of the IEEE/CVF Conference on Computer Vision and Pattern Recognition Workshops*, 24–25 (2020).

[27] Zeiler, M. D., Krishnan, D., Taylor, G. W. & Fergus, R. Deconvolutional networks. In *2010 IEEE Computer Society Conference on Computer Vision and Pattern Recognition*, 2528–2535 (IEEE, 2010).

[28] Springenberg, J. T., Dosovitskiy, A., Brox, T. & Riedmiller, M. Striving for simplicity: The all convolutional net. arXiv preprint arXiv:1412.6806 (2014).

[29] Vaswani, A. *et al.* Attention is all you need. In *Advances in Neural Information Processing Systems*, 5998–6008 (2017).

[30] Ravanelli, M. & Bengio, Y. Speaker recognition from raw waveform with sincnet. In *2018 IEEE Spoken Language Technology Workshop (SLT)*, 1021–1028 (IEEE, 2018).

[31] Samek W, Montavon G, Lapuschkin S, Anders CJ, Müller K-R (2021). Explaining deep neural networks and beyond: A review of methods and applications. Proc. IEEE.

[32] Tjoa, E. & Guan, C. A survey on explainable artificial intelligence (xai): Toward medical xai. *IEEE Trans. Neural Netw. Learn. Syst.* (2020). 10.1109/TNNLS.2020.3027314 33079674

[33] Kindermans, P.-J. *et al.* The (un) reliability of saliency methods. In *Explainable AI: Interpreting, Explaining and Visualizing Deep Learning*, 267–280 (Springer, 2019).

[34] Adebayo, J. *et al.* Sanity checks for saliency maps. arXiv preprint arXiv:1810.03292 (2018).

[35] Ma W (2021). A channel-mixing convolutional neural network for motor imagery EEG decoding and feature visualization. Biomed. Signal Process. Control.

[36] Borra D, Fantozzi S, Magosso E (2021). A lightweight multi-scale convolutional neural network for p300 decoding: Analysis of training strategies and uncovering of network decision. Front. Hum. Neurosci..

[37] Aellen FM, Göktepe-Kavis P, Apostolopoulos S, Tzovara A (2021). Convolutional neural networks for decoding electroencephalography responses and visualizing trial by trial changes in discriminant features. J. Neurosci. Methods.

[38] Ortega P, Faisal AA (2021). Deep learning multimodal fNIRS and EEG signals for bimanual grip force decoding. J. Neural Eng..

[39] Vilamala, A., Madsen, K. H. & Hansen, L. K. Deep convolutional neural networks for interpretable analysis of EEG sleep stage scoring. In *2017 IEEE 27th International Workshop on Machine Learning for Signal Processing (MLSP)*, 1–6 (IEEE, 2017).

[40] Farahat A, Reichert C, Sweeney-Reed CM, Hinrichs H (2019). Convolutional neural networks for decoding of covert attention focus and saliency maps for EEG feature visualization. J. Neural Eng..

[41] Zang B, Lin Y, Liu Z, Gao X (2021). A deep learning method for single-trial EEG classification in rsvp task based on spatiotemporal features of ERPs. J. Neural Eng..

[42] Vahid A, Mückschel M, Stober S, Stock A-K, Beste C (2020). Applying deep learning to single-trial EEG data provides evidence for complementary theories on action control. Commun. Biol..

[43] Wang, J. *et al.* A sequential graph convolutional network with frequency-domain complex network of EEG signals for epilepsy detection. In *2020 IEEE International Conference on Bioinformatics and Biomedicine (BIBM)*, 785–792 (IEEE, 2020).

[44] Jin X (2020). CTNN: A convolutional tensor-train neural network for multi-task brainprint recognition. IEEE Trans. Neural Syst. Rehabil. Eng..

[45] Petrosyan A, Sinkin M, Lebedev M, Ossadtchi A (2021). Decoding and interpreting cortical signals with a compact convolutional neural network. J. Neural Eng..

[46] Lawhern VJ (2018). EEGNet: A compact convolutional neural network for EEG-based brain-computer interfaces. J. Neural Eng..

[47] Haufe S (2014). On the interpretation of weight vectors of linear models in multivariate neuroimaging. Neuroimage.

[48] Sturm I, Lapuschkin S, Samek W, Müller K-R (2016). Interpretable deep neural networks for single-trial EEG classification. J. Neurosci. Methods.

[49] Ravindran, A. S. *et al.* Interpretable deep learning models for single trial prediction of balance loss. In *2020 IEEE International Conference on Systems, Man, and Cybernetics (SMC)*, 268–273 (IEEE, 2020).

[50] Ravindran AS (2022). Decoding neural activity preceding balance loss during standing with a lower-limb exoskeleton using an interpretable deep learning model. J. Neural Eng..

[51] Zhang X (2020). Adversarial representation learning for robust patient-independent epileptic seizure detection. IEEE J. Biomed. Health Inform..

[52] Gabeff V (2021). Interpreting deep learning models for epileptic seizure detection on EEG signals. Artif. Intell. Med..

[53] Ravindran AS (2019). Assaying neural activity of children during video game play in public spaces: A deep learning approach. J. Neural Eng..

[54] Schirrmeister RT (2017). Deep learning with convolutional neural networks for EEG decoding and visualization. Hum. Brain Map..

[55] Hartmann, K. G., Schirrmeister, R. T. & Ball, T. Hierarchical internal representation of spectral features in deep convolutional networks trained for EEG decoding. In *2018 6th International Conference on Brain-Computer Interface (BCI)*, 1–6 (IEEE, 2018).

[56] Mane, R., Robinson, N., Vinod, A. P., Lee, S.-W. & Guan, C. A multi-view CNN with novel variance layer for motor imagery brain computer interface. In *2020 42nd Annual International Conference of the IEEE Engineering in Medicine & Biology Society (EMBC)*, 2950–2953 (IEEE, 2020). 10.1109/EMBC44109.2020.9175874 33018625

[57] Li Y, Xiang J, Kesavadas T (2020). Convolutional correlation analysis for enhancing the performance of SSVEP-based brain-computer interface. IEEE Trans. Neural Syst. Rehabil. Eng..

[58] Thomas, A. H., Aminifar, A. & Atienza, D. Noise-resilient and interpretable epileptic seizure detection. In *2020 IEEE International Symposium on Circuits and Systems (ISCAS)*, 1–5 (IEEE, 2020).

[59] Ancona, M., Ceolini, E., Öztireli, C. & Gross, M. Towards better understanding of gradient-based attribution methods for deep neural networks. arXiv preprint arXiv:1711.06104 (2017).

[60] Kindermans, P.-J. *et al.* The (un)reliability of saliency methods. 1711.00867. (2017).

[61] Smilkov, D., Thorat, N., Kim, B., Viégas, F. & Wattenberg, M. Smoothgrad: removing noise by adding noise. arXiv preprint arXiv:1706.03825 (2017).

[62] Sharma, O. Deep challenges associated with deep learning. In *2019 International Conference on Machine Learning, Big Data, Cloud and Parallel Computing (COMITCon)*, 72–75 (IEEE, 2019).

[63] Paszke A, Wallach H (2019). Pytorch: An imperative style, high-performance deep learning library. Advances in Neural Information Processing Systems.

[64] Krol LR, Pawlitzki J, Lotte F, Gramann K, Zander TO (2018). SEREEGA: Simulating event-related EEG activity. J. Neurosci. Methods.

[65] Huang Y, Parra LC, Haufe S (2016). The New York head-a precise standardized volume conductor model for EEG source localization and tES targeting. NeuroImage.

[66] Mazziotta JC, Toga AW, Evans A, Fox P, Lancaster J (1995). A probabilistic atlas of the human brain: Theory and rationale for its development. Neuroimage.

[67] Marlin, A. Localization of cortical potentials evoked by balance disturbances. Master’s thesis (University of Waterloo, 2011).

[68] Marlin A, Mochizuki G, Staines WR, McIlroy WE (2014). Localizing evoked cortical activity associated with balance reactions: Does the anterior cingulate play a role?. J. Neurophysiol..

[69] Mierau A, Hülsdünker T, Strüder HK (2015). Changes in cortical activity associated with adaptive behavior during repeated balance perturbation of unpredictable timing. Front. Behav. Neurosci..

[70] Varghese JP, McIlroy RE, Barnett-Cowan M (2017). Perturbation-evoked potentials: Significance and application in balance control research. Neurosci. Biobehav. Rev..

[71] Yoo S-S, Lee J-H, O’Leary H, Panych LP, Jolesz FA (2008). Neurofeedback fMRI-mediated learning and consolidation of regional brain activation during motor imagery. Int. J. Imaging Syst. Technol..

[72] Lebon F, Horn U, Domin M, Lotze M (2018). Motor imagery training: Kinesthetic imagery strategy and inferior parietal fMRI activation. Hum. Brain Map..

[73] Mokienko O (2013). Increased motor cortex excitability during motor imagery in brain-computer interface trained subjects. Front. Comput. Neurosci..

[74] Kokhlikyan, N. *et al.* Captum: A unified and generic model interpretability library for pytorch . 2009.07896. (2020).

[75] Gildenblat, J. & contributors. Pytorch library for cam methods. https://github.com/jacobgil/pytorch-grad-cam (2021).

[76] Baehrens D (2010). How to explain individual classification decisions. J. Mach. Learn. Res..

[77] Hore, A. & Ziou, D. Image quality metrics: Psnr vs. ssim. In *2010 20th International Conference on Pattern Recognition*, 2366–2369 (IEEE, 2010).

[78] Brunet D, Vrscay ER, Wang Z (2011). On the mathematical properties of the structural similarity index. IEEE Trans. Image Process..

[79] Arras, L., Osman, A. & Samek, W. Ground truth evaluation of neural network explanations with clevr-xai. arXiv preprint arXiv:2003.07258 (2020).

[80] Nguyen, H. V. & Bai, L. Cosine similarity metric learning for face verification. In *Asian Conference on Computer Vision*, 709–720 (Springer, 2010).

